# Cerebrovascular risk factors and their time-dependent effects on stroke survival in the EMMA cohort study

**DOI:** 10.1590/1414-431X2023e12895

**Published:** 2023-09-22

**Authors:** A.C. Goulart, A.C. Varella, G. Tunes, A.P. Alencar, I.S. Santos, C. Romagnolli, T.E. Gooden, G.N. Thomas, G.Y.H. Lip, R.D. Olmos, P.A. Lotufo, I.M. Bensenor

**Affiliations:** 1 Centro de Pesquisa Clínica e Epidemiológica, Hospital Universitário, Universidade de São Paulo, São Paulo, SP, Brasil; 2 Instituto de Matemática e Estatística, Universidade de São Paulo, São Paulo, SP, Brasil; 3 Departamento de Medicina Interna, Faculdade de Medicina, Universidade de São Paulo, São Paulo, SP, Brasil; 4 Institute for Applied Health Research, University of Birmingham, Birmingham, United Kingdom; 5 Liverpool Centre for Cardiovascular Science, University of Liverpool and Liverpool Heart & Chest Hospital, Liverpool, United Kingdom; 6 Danish Center for Health Services Research, Department of Clinical Medicine, Aalborg University, Aalborg, Denmark

**Keywords:** Stroke epidemiology, Cerebrovascular risk factors, Stroke prevention

## Abstract

To investigate the time-dependent effects of traditional risk factors on functional disability in all-cause mortality post-stroke, we evaluated data from a long-term stroke cohort. Baseline cerebrovascular risk factors (CVRF) and functionality at 1 and 6 months were evaluated in survivors from a prospective stroke cohort using the modified Rankin scale (m-RS), which classifies participants as improvement of disability, unchanged disability (at least moderate), and worsening disability. Cox regression models considering baseline risk factors, medication use, and functionality 6 months after stroke were fitted to identify their time-dependent effects up to 12 years of follow-up. Adjusted hazard ratios (HR) with 95% confidence intervals (CI) are presented. Among 632 survivors (median age 68, 54% male, 71% first-ever episode), age and functional disability (unchanged and worsening) 6 months after ischemic stroke had time-dependent effects on all-cause mortality risk up to 12 years of follow-up. The most impacting risk factors were unchanged (at least moderate) (HR, 2.99; 95%CI: 1.98-4.52) and worsening disability (HR, 2.85; 95%CI: 1.26-6.44), particularly in the first two years after a stroke event (Time 1: ≥6 mo to <2.5 y). Worsening disability also impacted mortality in the period from ≥2.5 to <7.5 years (Time 2) of follow-up (HR, 2.43 (95%CI: 1.03-5.73). Other baseline factors had a fixed high-risk effect on mortality during follow-up. Post-stroke and continuous medication use had a fixed protective effect on mortality. Functional disability was the main contributor with differential risks of mortality up to 12 years of follow-up.

## Introduction

Traditionally, the analysis of risk factors and their effects in longitudinal epidemiological studies is based on fixed baseline data from a single time measurement, regardless of the length of follow-up. However, the effect of a risk factor on long-term mortality may vary over time, resulting in a weakening or strengthening of associations. In other words, even for risk factors measured only once (baseline), their effects can be strong in the long term but not necessarily in the short term, and vice-versa (1).

The proper analysis of effects over time should be driven by a clear research question. Two research questions, one for time-dependent effects (short-term versus long-term effects) of a fixed baseline risk factor and one for time-dependent risk factors (time-varying risk factors), can be analyzed with time-dependent Cox regression analysis. In this context, even sociodemographic determinants, especially low educational level, cerebrovascular risk factors, such as atrial fibrillation (AF), diabetes, hypertension, presence of multimorbidities, post-stroke depression, or disability, can have different impacts over time and might determine different mortality risks (2-7). Despite the importance of analyzing the real effect of risk factors on mortality, particularly in long-term prospective cohorts, most previous studies (2-7) demonstrated the effect of risk factors without considering time-stratified effects or time-dependent effects of fixed baseline risk factors (risk factors measured once, usually at baseline, considering effects analysis in different times during follow-up) or time-varying risk factors (risk factors measured multiple times during follow-up) (1).

Therefore, we aimed to evaluate time-dependent effects of traditional risk factors (measured at baseline) and post-stroke disability (measured 1 and 6 months after stroke) in the short- and long-term all-cause mortality in a Brazilian stroke cohort, the Study of Stroke Mortality and Morbidity (EMMA). EMMA is an ongoing longitudinal study that investigates the impact of cardiovascular risk factors and the role of secondary prevention on long-term mortality among individuals afflicted by stroke living in a low-middle socioeconomic income (LMIC) community in the city of São Paulo, Brazil.

## Material and Methods

### Study design and population

Study subjects were participants of the EMMA cohort, a well-characterized prospective long-term stroke surveillance cohort that has been ongoing since 2006 (8). The EMMA study was based on the Stepwise Approach to Stroke Surveillance (STEPS Stroke)-World Health Organization (WHO) (9,10). All patients older than 18 years with symptoms of acute stroke admitted to a single center, the Emergency Department (ED) of the HU-USP (University Hospital of the Universidade de São Paulo, Brazil), were invited to participate in the hospital phase of the EMMA study (8). The HU-USP is a secondary community hospital located in a low-income area of approximately 500,000 inhabitants on the west side of São Paulo city. Of note, all potential candidates for reperfusion therapies are regularly transferred to our referral center (Stroke Unit Care, Department of Neurology, Hospital das Clínicas of the Universidade de São Paulo), located five miles from the HU-USP. Thus, these cases were not included in these analyses. Further information about the EMMA study has been published elsewhere (8).

Between April 2006 and September 2014, 1,378 stroke cases (85.8% ischemic strokes [IS]) were enrolled in the EMMA cohort. For the present analyses, we excluded 195 hemorrhagic strokes, 304 participants with missing information on the modified Ranking Scale (mRS) at 1 and 6 months, 225 who died before the follow-up (6 months after stroke), and 22 without information on AF. Thus, the final sample comprised 632 participants.

Written informed consent was obtained from all EMMA participants or their legal representatives/caregivers (usually a close family member). The study was approved by the HU-USP Ethical Committee (# 593/05).

### EMMA data collection

All data collection was performed by a trained interviewer (nurse) at hospital admission (personal interview) at 1 and 6 months (telephone interview) according to the WHO STEPS Stroke Manual instructions. Quality control was assured for cross-checking information, which was done by three medical coordinators of the EMMA study (8,9). As an extension of WHO STEPS stroke, we evaluated EMMA participants yearly or until death for up to 12 years from baseline. All losses before 12 years were censored on the date they were last contacted or on which information about their vital status (alive) was confirmed by telephone contact or electronic hospital registers. Mortality data were confirmed by official death certificates in collaboration with the city of São Paulo's health statistics system (PRO-AIM, Program for Improvement of Mortality Information in the Municipality of São Paulo), State Health Offices (SEADE Foundation, São Paulo State Healthcare Data Analysis System), and the Brazilian Ministry of Health.

### Stroke definition

Stroke was defined according to WHO criteria as “a focal (or at times global) neurological impairment of sudden onset lasting more than 24 h (or leading to death) of presumed vascular origin. This clinical definition has, therefore, four components: 1) a neurological impairment or deficit; 2) sudden onset; 3) lasting more than 24 h (or leading to death); and 4) of presumed vascular origin” (9). In addition to these four components, stroke diagnosis was validated by a medical doctor from the study and supported by no-contrast computed tomography (CT) performed within 24-48 of hospital admission for inclusion as a stroke case in the EMMA study (8). Furthermore, stroke diagnosis was classified according to the International Classification of Diseases, 10th Edition (ICD-10: I60-I63.9). Here, we analyzed individuals with IS (code I63.X). Of note, history of stroke was based on information from the patient, caregiver, or hospital records.

### Sociodemographic and clinical data

At baseline (hospital admission due to stroke), we collected information about socio-demographic factors (age, education, marital status, and race as self-reported skin color: white, mixed, black, and yellow), stroke episode (first-ever or recurrent stroke), pre-existing cerebrovascular risk factors (CVRF) such as smoking, alcohol consumption, and co-morbidities such as hypertension, diabetes mellitus, dyslipidemia, heart failure, coronary heart disease, and atrial fibrillation (AF). Data about clinical co-morbidities (before index event) and cardiovascular medication were validated by two senior medical researchers, based on medical registries. We considered all clinical diagnoses based on medical history and/or the use of medications. Diagnosis of AF at baseline was defined by ECG tracings (before or at hospital admission). Data on medication use such as anti-hypertensive, lipid-lowering, anti-diabetic, anti-platelet, and anti-coagulants were collected at hospital admission (study baseline) and 1 month after hospital discharge due to stroke. The degree of functional disability was evaluated by the modified Rankin Scale (mRS) at 1 and 6 months after stroke. Stroke functionality by mRS was classified as improvement of disability, unchanged disability (at least moderate), or worsening disability at 6 months after stroke compared to 1 month after stroke. In brief, the m-RS ranges from 0 to 6, with higher scores indicating greater disability and 0-2 is generally considered a good outcome with individuals assuming complete functional independence and 6 corresponds to an individual who is deceased (11,12). For the present analysis, we considered only survivors 6 months after the index event, thus, we reported the m-RS evaluation from 0 to 5 points at 1 and 6 months.

### Statistical analysis

Categorical variables were analyzed by the chi-squared test and reported as absolute and relative frequencies. As continuous variables did not have a normal distribution, they were analyzed by the Wilcoxon test and reported as median values with respective interquartile ranges (IQR). Sociodemographic and clinical data and use of medication for clinical chronic conditions (anti-hypertensive, anti-diabetic, lipid-lowering medication, anti-platelet, and anti-coagulants) at hospital admission due to stroke, as well as functional status by mRS (at 1 and 6 months) were analyzed by sex.

For the all-cause mortality outcome, we calculated hazard ratios (HR) with respective 95% confidence intervals (CIs) derived from multivariable models adjusted by potential predictors: age, sex, education attainment, marital status, hypertension, diabetes, dyslipidemia, smoking, recurrent stroke (at baseline), functional disability up to 6 months, and medication use according to index event (pre-stroke, post-stroke, and continuous use).

Kaplan-Meier survival curve (13) and Cox regression models (14) were fitted to the data. We first fitted usual Cox models that did not include time-dependent effects. Analysis of Schoenfeld residuals and hypothesis testing for proportional hazards indicated that hazards were not proportional for the variables post-stroke functionality (P-value=0.011) and age (P-value=0.035) (15). We then included time-dependent effects for these two variables and time change points of effects were chosen based on the plot of Schoenfeld residuals. Since Schoenfeld residuals reveal the functional form of the time-dependent effect, if the tendency line of the plot is close to a horizontal line, then there is evidence that a usual Cox model is appropriate; however, the lines in plots were very distant from a horizontal straight line and they suggested that a reasonable approximation of this time-dependent effect would be a step-function with three breaks: Time 1: ≥6 months to <2.5 years, Time 2: ≥2.5 to <7.5 years, and Time 3: ≥7.5 years or longer (Supplementary Figure S1)

Statistical analyses were performed with the statistical software SPSS version 27.0 (IBM, USA) and the R software (R Core Team). For all analyses, P-values less than 0.05 were considered significant.

## Results

**Figure 1:**
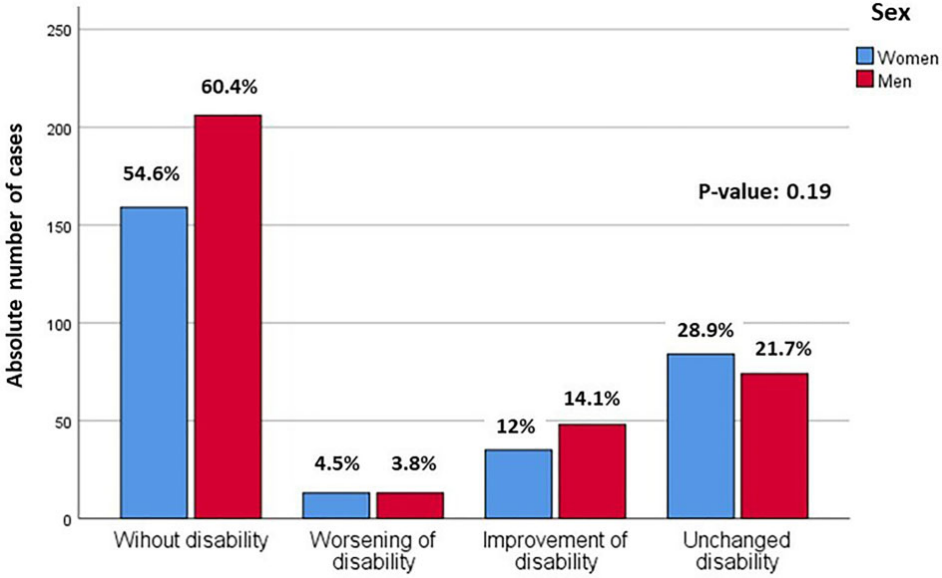
Functionality by the modified Ranking scale at 6 months after stroke in the Study of Stroke Mortality and Morbidity (EMMA) cohort.

In the 632 IS survivors (median age 68 years; 71% first-ever stroke, 54% male) investigated, we found high frequencies of CVRF such as hypertension (94.1%), diabetes (58.1%), dyslipidemia (61.1%), and smoking (current 30.7%, past 14.1%) and relatively low continuous use of medications for controlling these risk factors (49.8%) (Table 1). Most individuals in this sample (57.8%) had mild or no disability; however, 25% had no improvement in their functionality (at least moderate) since the acute event, regardless of sex (Figure 1).

**Table 1:** Baseline characteristics of the 632 participants from the Study of Stroke Mortality and Morbidity (EMMA) study.

	Men (n=341)	Women (n=291)	Total (n=632)	P-value
Sociodemographic				
Median age, years (IQR)	66 (56-75)	71 (59-78)	68 (57-77)	0.003
Race (skin color*, n (%)				0.23
White	207 (61.2)	177 (60.8)	384 (61.0)	
Brown	97 (28.7)	95 (32.6)	192 (30.5)	
Black	26 (7.7)	17 (5.8)	43 (6.8)	
Yellow	8 (2.4)	2 (0.7)	10 (1.6)	
Education, n, %				<0.0001
Illiterate	39 (11.4)	67 (23.0)	106 (16.8)	
1-7 years	167 (49.0)	140 (48.1)	307 (48.6)	
≥8 years	135 (39.6)	84 (28.9)	219 (34.7)	
Marital status, n (%)				<0.0001
Not married	33 (9.7)	50 (17.3)	83 (13.2)	
Married	236 (69.4)	118 (40.8)	354 (56.3)	
Divorced	29 (8.5)	22 (7.6)	51 (8.1)	
Widowed	40 (11.8)	97 (33.6)	137 (21.8)	
Ignored	2 (0.6)	2 (0.7)	4 (0.6)	
Comorbidities				
Number of chronic comorbidities, n (%)				0.69
0	6 (1.8)	4 (1.4)	10 (1.6)	
1-2	131 (38.4)	121 (41.6)	252 (39.9)	
≥3	204 (59.8)	166 (57.0)	370 (58.5)	
First-ever stroke, n (%)	233 (68.3)	216 (74.2)	449 (71.0)	0.10
Hypertension, n (%)	319 (93.5)	276 (94.8)	595 (94.1)	0.49
Atrial fibrillation, n (%)	41 (12.0)	52 (17.9)	93 (14.7)	0.04
Diabetes, n (%)	205 (60.1)	162 (55.7)	367 (58.1)	0.26
Dyslipidemia, n (%)	208 (61.0)	178 (61.2)	386 (61.1)	0.97
Heart failure, n (%)	90(26.4)	66 (22.7)	156 (24.7)	0.28
Chronic kidney disease, n (%)	81 (23.8)	36 (12.4)	117 (18.5)	<0.0001
Coronary artery disease, n (%)	71 (20.9)	55 (18.9)	126 (20.0)	0.54
COPD, n (%)	18 (5.3)	11 (3.8)	29 (4.6)	0.37
Smoking, n (%)				<0.0001
Never	153 (44.9)	196 (67.4)	349 (55.2)	
Current	126 (37.0)	68 (23.4)	194 (30.7)	
Past	62 (18.2)	27 (9.3)	89 (14.1)	
Medication use, n (%)				0.18
Never	41(12.0)	35 (12.0)	76 (12.0)	
Pre-stroke	48 (14.1)	54 (18.6)	102 (16.1)	
Post-stroke	85 (24.9)	54 (18.6)	139 (22.0)	
Continuous use	167 (49.0)	148 (50.9)	315 (49.8)	

* Three male patients had no defined self-reported skin color (race). Never used: Patients who never used pre- and post-stroke medications for chronic clinical conditions (hypertensives and/or antidiabetic and/or lipid-lowering drugs and/or antiplatelets and/or anticoagulants); Pre-stroke: Patients who only used pre-stroke medications for chronic clinical conditions; Post-stroke: Patients who only used post-stroke medications for chronic clinical conditions; Continuous use: Patients kept using medications (pre- and post-stroke) for chronic clinical conditions. IQR: Interquartile range; COPD: Chronic obstructive pulmonary disease. P-values were derived from the Mann-Whitney test for continuous variables and chi-squared was used for categorical variables.

**Figure 2:**
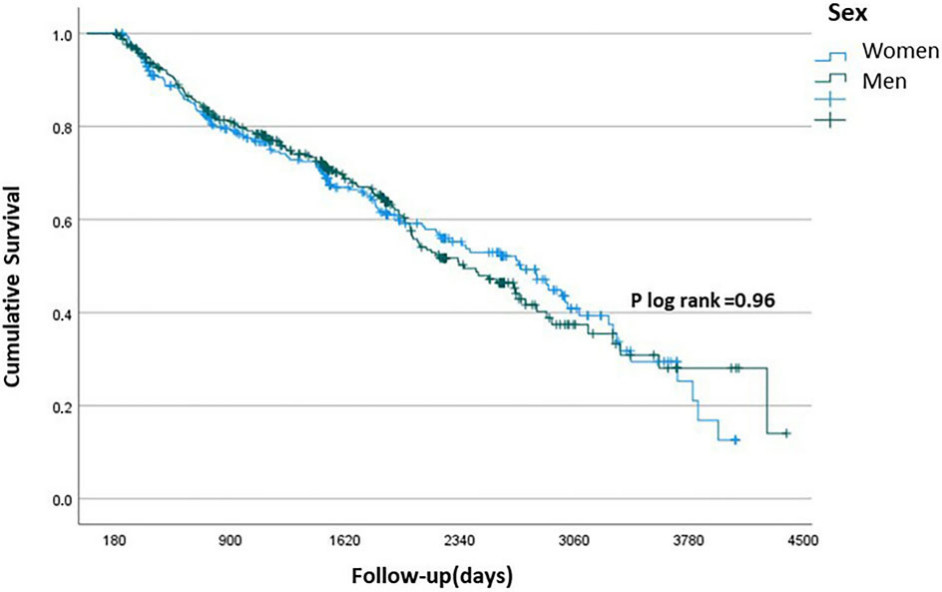
Kaplan Meyer survival curve for all-cause mortality by sex among ischemic stroke patients during the 12-year follow-up in the Study of Stroke Mortality and Morbidity (EMMA) cohort.

**Figure 3:**
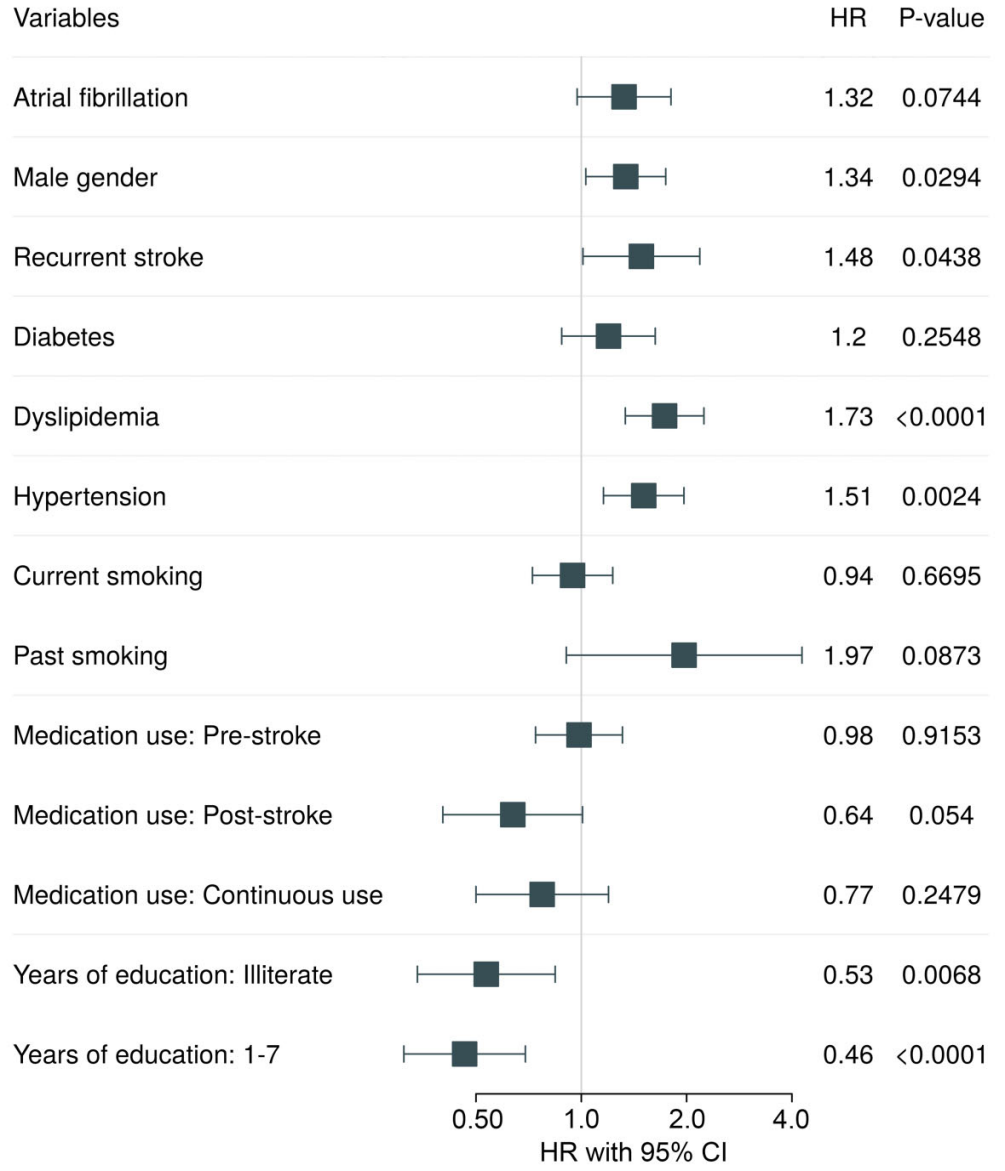
Fixed baseline risk factors without time-dependent effect on all-cause mortality in the Study of Stroke Mortality and Morbidity (EMMA) cohort.

**Figure 4:**
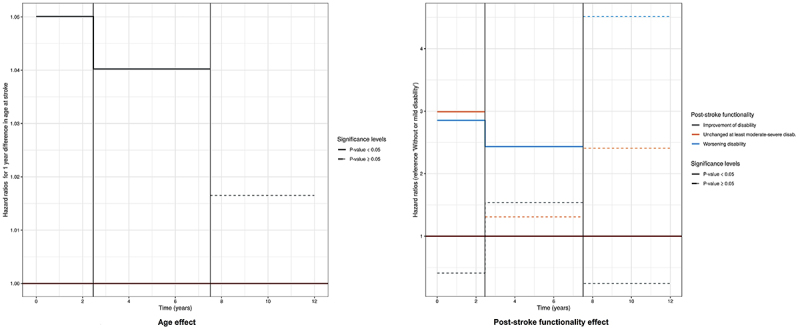
Fixed baseline risk factors with time-dependent effect on all-cause mortality in the Study of Stroke Mortality and Morbidity (EMMA) cohort.

Among 275 deaths attested along the follow-up, 22.5, 32, and 45.5% were reported as cerebrovascular, cardiovascular, and non-cardiovascular basic causes, respectively. About 56.5% (357/632) of stroke participants survived up to the 12 years of follow-up. Mortality did not differ between sexes, with P log rank=0.96 (Figure 2). Of all risk factors, age and functional disability (unchanged and worsening) had time-dependent effects on mortality risk. However, the most impacting risk factor was functional disability, particularly in the first two years (Time 1: ≥6 months to <2.5 years) after the stroke event; HR=2.99 (95%CI: 1.98-4.52) for unchanged disability (at least moderate) and HR=2.85 (95%CI: 1.26-6.44) for worsening disability. Worsening disability also impacted mortality in Time 2 (≥2.5 to <7.5 years of follow-up, HR=2.43 (95%CI: 1.03-5.73)). After Time 3 (≥7.5 years or longer), we found no evidence of an effect of age and disability on long-term mortality. Sociodemographic factors such as male gender, illiteracy, as well as some cerebrovascular risk factors as AF (HR=1.32; 95%CI: 0.97-1.80), diabetes (HR=1.51, 95%CI: 1.16-1.97), and recurrent stroke (HR=1.73; 95%CI: 1.34-2.24) had fixed high-risk effects on mortality up to 12 years of follow-up. On the other hand, the use of medications post-stroke (HR=0.53; 95%CI: 0.34-0.84) and continuous medication use (HR=0.46; 95%CI: 0.31-0.69) had a fixed protective effect on mortality (Figures 3 and 4, Tables 2 and 3).

**Table 2:** Hazard ratios (HR) of all-cause mortality for fixed baseline risk factors without time-dependent effect along 12-y follow-up in the Study of Stroke Mortality and Morbidity (EMMA) cohort.

	HR (95%CI)	P-value
Male gender	1.34 (1.03-1.74)	0.03
Educational level		
Illiterate	1.49 (1.01-2.18)	0.04
1-7 years of education	1.20 (0.88-1.63)	0.25
Atrial fibrillation	1.32 (0.97-1.80)	0.07
Recurrent stroke	1.73 (1.34-2.24)	<0.0001
Diabetes	1.51 (1.16-1.97)	0.002
Dyslipidemia	0.94 (0.73-1.23)	0.67
Hypertension	1.97 (0.91-4.27)	0.08
Smoking		
Current	0.98 (0.74-1.31)	0.92
Past	0.64 (0.40-1.01)	0.054
Medication use		
Pre-stroke	0.77 (0.50-1.19)	0.25
Post-stroke	0.53 (0.34-0.84)	0.007
Continuous use	0.46 (0.31-0.69)	<0.0001

Multivariable model included the adjustments for age, sex, educational level, marital status, hypertension, diabetes, dyslipidemia, smoking, recurrent stroke, functional disability up to 6 months, and medication use according to index event (pre-, post-stroke, and continuous use).

**Table 3:** Hazard ratios (HR) of all-cause mortality for fixed baseline risk factors with time-dependent effect along 12-y follow-up in the Study of Stroke Mortality and Morbidity (EMMA) cohort.

	Time 1 (≥6 months to <2.5 years)		Time 2 (≥2.5 to <7.5 years)		Time 3 (≥7.5 years or longer)	
	HR (95%CI)	P-value	HR (95%CI)	P-value	HR (95%CI)	P-value
Age*	1.05 (1.03-1.07)	<0.0001	1.04 (1.02-1.06)	<0.0001	1.02 (0.98-1.059)	0.43
Post-stroke functionality						
Improvement of disability	0.41 (0.16-1.05)	0.06	1.54 (0.97-2.43)	0.07	0.25 (0.03 -2.09)	0.20
Unchanged disability	2.99 (1.98-4.52)	<0.0001	1.31 (0.85-2.01)	0.22	2.41 (0.91-6.39)	0.08
Worsening of disability	2.85 (1.26-6.44)	0.01	2.43 (1.03-5.73)	0.04	4.52 (0.55-36.80)	0.16

* Hazard ratio for 1-year difference in age at stroke. Multivariable model included adjustments for age, sex, educational level, marital status, hypertension, diabetes, dyslipidemia, smoking, recurrent stroke, functional disability up to 6 months, and medication use according to index event (pre-, post-stroke, and continuous use). Levels of functionality by modified Rankin scale (m-RS) at 1 month and 6 months after stroke: Improvement of disability at any level (from 0 to 5, except 6=death), unchanged disability at least moderate (≥3), and worsening of disability at any level (from 0 to 5, except 6=death).

## Discussion

This study aimed to investigate the time-dependent effect of main traditional risk factors on all-cause mortality during 12 years of follow-up. Our main findings revealed the different impacts of those risk factors on all-cause mortality over time. Male gender, illiteracy, previous history of stroke (recurrent stroke at baseline), and diabetes were associated with increased, but fixed hazard ratios of long-term mortality. On the other hand, the use of medication to control traditional CVRF (post-stroke use and continuous use) caused a reduction in the risk of mortality by 50% up to 12 years of follow-up. Age and post-stroke disability were the only risk factors with time-dependent effects on mortality mainly between 6 months and two and a half years after stroke. Compared to those without disability, individuals who maintained or worsened their disability 6 months after the index event had almost three times the risk of death in the first two years after stroke.

Previously published articles, including data reported from the EMMA cohort (2,4-6), showed increased risk of mortality among those subjects with low education, cardiovascular risk factors, and post-stroke depression. Even though those previous studies did not account for risk factors' time-dependent effects, our study supports some of these findings. Here, low education, diabetes, male sex, and recurrent stroke were found to have the same positive association with mortality during the entire follow-up period (12 years).

Regarding functional disability, studies have found a significant association between low level of education and high level of disability, as well as post-stroke disability and mortality (7,16). Our results expand these findings by showing the influence of time on the effect of disability on mortality, regardless of educational attainment. We found a stronger association between functional disability and mortality shortly after stroke onset (from 6 months up to 2.5 years).

The dependent effect of age in our study was evaluated for a 1-year difference in age at the index event, and the increased HR in the risk of dying was mainly verified in the first two years after stroke (Time 1). In previous analyses from the EMMA cohort, where follow-up data was analyzed in years 1, 2, 3, and 4 after stroke, age was a strong predictor of mortality, with HR around 3 times among those aged 80 years or more (5). In the latter method, time of follow-up was stratified, but the changes in the dependent effect of age on long-term mortality were not considered. In both analyses (previous and current) mortality was calculated by Cox regression models, but in the present analyses, the measurement of the time-dependent effect was more accurate in quantifying the influence of each risk factor over time than simply considering different follow-up periods.

Moreover, studies have found medication use for traditional CVRF to have an important protective effect against mortality in stroke patients (17-19). This study also supports those findings. When analyzing medication use after stroke and continuous medication use (including pre-stroke), we found a strong inverse association with all-cause mortality after 12 years. Medication use had a fixed and constant effect during the entire follow-up period.

To the best of our knowledge, no prospective stroke cohorts have investigated the time-dependent effects of baseline risk factors on long-term mortality. In a different scenario, Bellera et al. evaluated the varying effects of risk factors on mortality among women with breast cancer, showing that traditional Cox regression might mask the effects of certain variables in the short-term versus long-term follow-up (20). They found that a particular risk factor increased the risk of metastases early on, becoming protective afterward. Their findings make evident the importance of accounting for the time-dependent effects of baseline variables, especially as the cohort ages during follow-up years. Other previous studies with stroke populations used different methodology to evaluate the time-varying risk of some selected risk factors (21,22). In the study of Huang et al. (21), the time-varying effects of psychological distress on the functional recovery of stroke patients were analyzed. Participants were interviewed five times after stroke onset (from 5 days up to 6 months). They reported a significant effect of psychological distress on functional recovery over time but did not analyze its effects on mortality.

In the study of Liu et al. (22), risk factors associated with functional recovery and psychological distress in stroke patients were evaluated using 3 different measurements in time, and it was demonstrated that the National Institutes of Health Stroke Scale scores (NIHSS) and depression levels affected functional recovery. Also, depression was associated with anxiety, low social support levels, and low education. The main conclusions of this study revealed the importance of early poststroke rehabilitation with 50% of patients with full functional recovery after 6 months, but the effect of time-varying risk factors on mortality was not included in the analyses of Liu et al. (22). In our study, although we evaluated mostly variables measured at baseline, we also found that functional disability should be addressed as early as possible due to its stronger association with mortality in the first 2 years.

### Strengths and limitations

The current research corroborates the knowledge gap about the different effects that risk factors can have over time, even when measured only at baseline. Of note, the classical Cox model relies on the proportional hazard's assumption, implying that the factors have a constant impact on the hazard - or risk - over time (14). The present study had a robust validation process of stroke cases, including a collection of data on all cerebrovascular risk factors and medications (before and during follow-up after an index event), entirely supervised by the medical research team.

This study had some limitations, most of them related to selection bias. The EMMA cohort is based on data from a single center that is a secondary community hospital without specialized treatment options (e.g., thrombolysis/thrombectomy). Although patients with IS who report any acute therapy are usually transferred to our reference (tertiary) hospital, we cannot rule out a selection bias from a highly selected segment of stroke patients excluded from the EMMA cohort that limits the external generalization of our data.

Although we did not have NIHSS or different measures of disability at hospital admission and even stroke recurrence data during follow-up, we measured mRS at two points during follow-up (1 and 6 months after stroke). This information about disability after stroke allowed us to calculate the evolution of functionality (unchanged, improved, and worsened) to better evaluate the time-dependent effect of this relevant factor associated with both short- and long-term mortality.

Although most individuals had mild or no disability, which could influence the magnitude of our findings by underestimating the impact of functional disability on long-term mortality, we were able to report important data about higher levels of functional disability at 6 months related to higher mortality during follow-up. This data is particularly important for communities with low-middle socioeconomic status and limited resources for rehabilitation after stroke (23).

In addition, most individuals who are disabled after stroke tend to have more difficulty of locomotion to receive proper care and achieve a good recovery. In the present analysis, we found about 8.3% of functional deterioration six months after stroke. We speculated that the lack of rehabilitation after hospital discharge could have contributed to this worsening. About 45.7% of participants reported some kind of rehabilitation (physiotherapy and speech therapy) after their stroke event, however we did not know the reasons for the low rehabilitation adherence in the EMMA cohort.

Finally, an observational study design does not allow us to rule out the possibility of immortal time bias in our analyses (24,25).

### Conclusions

Functional disability was the main contributor to short- and long-term post-stroke mortality. Male sex, low education attainment, and traditional baseline CVRF such as diabetes, AF, and recurrent stroke had a fixed high-risk effect on mortality up to 12 years after the acute event. Medication use, particularly the continuous use of medications for CVRF, more than halved the risk of death throughout the 12-year follow-up period. Thus, the present study reinforces tertiary prevention by early rehabilitation among those with post-stroke functional disability and secondary prevention by the use of medications for controlling risk factors and thus prevent recurrent strokes and reduce mortality risk after stroke.

## Supplementary Material

Click here to view [pdf].
